# Hypoxia-induced DNA hypermethylation in human pulmonary fibroblasts is associated with Thy-1 promoter methylation and the development of a pro-fibrotic phenotype

**DOI:** 10.1186/1465-9921-13-74

**Published:** 2012-08-31

**Authors:** Claire M Robinson, Roisin Neary, Ashleigh Levendale, Chris J Watson, John A Baugh

**Affiliations:** 1 UCD School of Medicine & Medical Science, UCD Conway Institute, University College Dublin, Dublin 4, Ireland

**Keywords:** Hypoxia, Hypermethylation, Thy-1, Lung fibrosis, Myofibroblast

## Abstract

**Background:**

Pulmonary fibrosis is a debilitating and lethal disease with no effective treatment options. Understanding the pathological processes at play will direct the application of novel therapeutic avenues. Hypoxia has been implicated in the pathogenesis of pulmonary fibrosis yet the precise mechanism by which it contributes to disease progression remains to be fully elucidated. It has been shown that chronic hypoxia can alter DNA methylation patterns in tumour-derived cell lines. This epigenetic alteration can induce changes in cellular phenotype with promoter methylation being associated with gene silencing. Of particular relevance to idiopathic pulmonary fibrosis (IPF) is the observation that Thy-1 promoter methylation is associated with a myofibroblast phenotype where loss of Thy-1 occurs alongside increased alpha smooth muscle actin (α-SMA) expression. The initial aim of this study was to determine whether hypoxia regulates DNA methylation in normal human lung fibroblasts (CCD19Lu). As it has been reported that hypoxia suppresses Thy-1 expression during lung development we also studied the effect of hypoxia on Thy-1 promoter methylation and gene expression.

**Methods:**

CCD19Lu were grown for up to 8 days in hypoxia and assessed for global changes in DNA methylation using flow cytometry. Real-time PCR was used to quantify expression of Thy-1, α-SMA, collagen I and III. Genomic DNA was bisulphite treated and methylation specific PCR (MSPCR) was used to examine the methylation status of the Thy-1 promoter.

**Results:**

Significant global hypermethylation was detected in hypoxic fibroblasts relative to normoxic controls and was accompanied by increased expression of myofibroblast markers. Thy-1 mRNA expression was suppressed in hypoxic cells, which was restored with the demethylating agent 5-aza-2^′^-deoxycytidine. MSPCR revealed that Thy-1 became methylated following fibroblast exposure to 1% O_2_.

**Conclusion:**

These data suggest that global and gene-specific changes in DNA methylation may play an important role in fibroblast function in hypoxia.

## Background

Idiopathic pulmonary fibrosis (IPF) is a progressive and lethal fibrotic lung disease of unknown aetiology that affects approximately 5 million people worldwide [1]. The disease is associated with progressive scarring of the lung that subsequently results in loss of lung function. Incidence of IPF appears to be rising. Recent data from the UK show a 35% increase in the incidence of IPF in the primary care setting between 2000 to 2008, with an overall incidence rate of 7.44 per 100,000 person-years, and an estimate of over 5,000 new cases diagnosed per year [2]. There are no effective therapeutic strategies for the treatment of IPF and patients have a very poor prognosis with a median survival of 2-3 years [3,4]. There is subsequently a great need for increased comprehension of disease pathogenesis and the identification of novel therapeutics.

IPF is characterized by non-resolving injury to the epithelial cells that line the lung [5]. Alveolar epithelial cell injury results in the release of cytokines and growth factors, such as transforming growth factor beta (TGFβ). Whilst inappropriate epithelial responses may initiate the injurious process in the lung, it is the activated fibroblasts (myofibroblasts) that produce the scar tissue that ultimately abolishes lung function. As well as the pro-fibrotic cytokine milieu there is increasing interest in other local tissue environmental factors that may contribute to myofibroblast activation. Fibroblast proliferation, inflammatory cell infiltration and interstitial thickening potentially combine with alveolar ventilation defects to increase the consumption and limit the supply of oxygen to the injured lung resulting in local tissue hypoxia. Pulmonary tissue oxygen levels are hard to quantify but a role for hypoxia in the progression of pulmonary fibrosis has been supported by the observation that there is increased stabilisation of the hypoxia-inducible transcription factor (HIF-1α) in lung tissue taken from patients with IPF as well as in mice with bleomycin-induced pulmonary fibrosis [6]. Whilst a great deal is known about the role of transcription factors like HIF-1α in adaptive responses to acute hypoxia, the mechanisms that regulate transcriptional events in response to chronic hypoxia are less well understood.

We and others have shown that hypoxia regulates DNA methylation in cancer-derived cell lines [7-9]. DNA methylation is an epigenetic mechanism whereby a cytosine residue gains a methyl (CH_3_) group on the 5^th^ carbon of the pyramidine ring to become 5 methylcytosine (5MeC). This process is catalysed by a group of enzymes called the DNA methyltransferases (DNMTs). Increased methylation in regions known as CpG islands of gene promoters is associated with transcriptional repression [10]. Given the importance of DNA methylation in regulating chromatin condensation and gene expression we propose that this epigenetic mechanism is crucial for cellular adaptation to hypoxic conditions. In the case of IPF we hypothesised that this is important in regulating fibroblast phenotype during disease progression.

In support of this hypothesis is an increasing body of evidence that implicates an altered global DNA methylation profile in IPF pathogenesis [11,12]. It is of particular interest that DNA hypermethylation has been associated with myofibroblast differentiation [13,14]. Recent studies have demonstrated the contribution of several gene-specific silencing events mediated by DNA hypermethylation as promoting myofibroblast differentiation as well as promoting their persistence in fibrotic pulmonary disease [13,15,16]. One such gene is thymocyte differentiation antigen-1 (Thy-1). Thy-1 is a membrane bound glycoprotein. It is an important regulator of cell-cell and cell-matrix interactions and its expression can also affect intracellular signalling pathways [17,18]. Thy-1 is expressed on a variety of cell types including thymocytes, T cells and neuronal cells as well as on subsets of fibroblasts [19]. Thy-1 (-ve) cells are associated with a myofibroblast phenotype [20-22]. In pulmonary myofibroblasts Sanders et al. have demonstrated that Thy-1 loss is due to epigenetic regulation of the gene [15,23]. Thy-1 was shown to be hypermethylated in fibroblastic foci of lung tissue taken from patients with IPF, where methylation of the Thy-1 gene inhibited its expression [15]. Despite the fact that epigenetic regulation of Thy-1 has been reported as regulating its expression [15,23], there is currently limited understanding of what environmental factors govern the availability of Thy-1 (+ve) and Thy-1(-ve) cells. Interestingly it has been demonstrated that Thy-1 expression is suppressed in lungs of hypoxic mice and this is associated with increased TGFβ1 activation [24]. Whether this suppression was due to increased DNA methylation in the Thy-1 promoter was not reported.

Given the results from these studies investigating Thy-1 expression and regulation, along with our previous findings that hypoxia can impact a cells DNA methylation profile, the aim of this study was to investigate hypoxic regulation of DNA methylation in commercially available normal human pulmonary fibroblasts. We hypothesised that hypoxic regulation of DNA methylation promotes myofibroblast differentiation and that hypoxia induced methylation of Thy-1 is an important component of this process.

## Methods

### Cell culture

Commercially available normal human pulmonary fibroblasts (CCD19Lu) were acquired from American Type Culture Collection. Cells were cultured in minimum essential medium (MEM; Sigma) supplemented with 10% foetal calf serum (FCS; Gibco), 2 mM L-glutamine (Gibco) and penicillin/streptomycin (Gibco) in a 5% CO_2_ humidified incubator kept at 37°C. When a chronic hypoxia environment was required, a 1% oxygen (O_2_) atmosphere was created using a hypoxic chamber (Coy Laboratories). Cells were grown for up to 8 days in 1% oxygen (5% CO_2_).

### Treatments

For demethylation analysis, cells were treated with 1 μM 5-aza-2^′^-deoxycytidine (5-aza2dC; Sigma) and replenished every second day for 8 days.

### Flow cytometry

Cells were harvested by trypsinization and fixed in Carnoy’s Solution (3:1 methanol/acetic acid). Immuno-staining was then conducted using a monoclonal anti-5MeC (Eurogentec) antibody. Prior to staining with anti-5MeC cells were pre-incubated with 1 M HCl at 37°C for 1 h. IgG1 negative controls were used at equivalent concentrations as the primary antibody. Antibody binding was visualised using fluorescein isothiocyanate isomer 1 (FITC) conjugated rabbit anti-mouse secondary antibody (Dako) and analysed using a CYAN flow cytometer. Results were assessed using SUMMIT software (Dako).

### Quantitative real-time PCR (QPCR)

RNA was isolated from cells with NucleoSpin RNA II Kit (Macherey-Nagel) prior to first strand cDNA synthesis using SuperScript II RT (Invitrogen). QPCR primers were designed so that one of each primer pair was exon/exon boundary spanning to ensure only mature mRNA was amplified. The sequences of the gene-specific primers used are as follows;

α-SMA; 5^′^-CGTTACTACTGCTGAGCGTGA-3^′^ (forward), 5^′^-AACGTTCATTTCCGATGGTG 3'(reverse) collagen type I (α1); 5^′^- GAACGCGTGTCATCCCTTGT-3^′^(forward), 5^′^ -GAACGAGGTAGTCTTTCAGCAACA -3^′^(reverse) collagen type III (α1); 5^′^- AACACGCAAGGCTGTGAGACT -3^′^(forward), 5^′^- GCCAACGTCCACACCAAATT -3^′^(reverse) Thy-1; 5^′^- ATGAACCTGGCCATCAGTCT-3^′^ (forward), 5^′^-CACGTGCTTCTTTGTCTCA-3^′^ (reverse).

Following extensive quantification of various potential housekeeping genes in normoxia and hypoxia, the expression of beta-2-microglobulin (B2M) was found to be unaffected by experimental conditions (data not shown) and was therefore deemed an appropriate housekeeper. The sequences for B2M were as follows: 5^′^- AGGCTATCCAGCGTACTCCA-3^′^ (forward), 5^′^-CCAGTCCTTGCTGAAAGACA-3^′^ (reverse).

QPCR was performed using Platinum SYBR Green QPCR SuperMix-UDG (Invitrogen). Amplification and detection were carried out using Mx3000P System (Stratagene). The PCR cycling program consisted of 40 three-step cycles of 15 s/95°C, 30 s/T_A_ and 30 s/72°C. Each sample was amplified in duplicate. In order to confirm signal specificity, a melting program was carried out after the PCR cycles were completed. The samples were quantified using the delta delta CT method.

### Methylation specific PCR

The methylation status of Thy-1 was investigated using Methylation Specific PCR (MSPCR). Total genomic DNA was isolated from normoxic and hypoxic CCD19Lu cells using DNeasy DNA extraction kit (Qiagen), according to manufacturer's instructions. Prior to MSPCR, 1 μg of DNA was bisulfite-treated using the EZ DNA Methylation kit (Zymo Research) according to manufacturer's instructions. Previously published Thy-1 MSPCR primers were used to discriminate between methylated and unmethylated DNA [15]; methylated forward, 5^′^-TATTTTTATATTAATGCGGGATCGT-3^′^, methylated reverse, 5^′^-CGATTACTACACCCAACTCGAA-3^′^, unmethylated forward, 5^′^-TTATTTTTATATTAATGTGGGATTGT-3^′^, unmethylated reverse 5^′^-TCCAATTACTACACCCAACTCAAA -3^′^. MSPCR products were visualised by electrophoresis on 2% SYBRsafe stained agarose gels.

### Western blot

α-SMA expression was measured in normoxic and hypoxic CCD19Lu. Whole cell protein extractions were performed using radioimmuoprecipitation buffer (RIPA buffer, Millipore) with a 1 × protease inhibitor cocktail (Complete Mini, Roche). All proteins were quantified and subsequently normalised using a BCA kit (Pierce). Polyacrylamide gels were used to separate proteins by size and they were then transferred onto a polyvinylidene fluoride **(**PVDF**)** membrane (Immobilion-P transfer membrane, Millipore). Following a blocking step in 5% milk, the membranes were incubated overnight at 4°C in primary antibody directed towards α-SMA (mouse anti αSMA; Sigma). After washing, the membranes were then incubated in a HRP conjugated goat anti mouse secondary antibody (Santa Cruz). They were then washed and developed. α-tubulin (mouse anti α tubulin; Cedarlane) was used as a loading control.

### Immunocytochemistry

CCD19Lu were seeded onto wells of coverslides. Following appropriate treatments, the cells were washed in PBS and then fixed and permeabilised using 70% methanol. After blocking, the slides were incubated in primary antibody (mouse anti α-SMA; Sigma) for 1 h at room temperature. They were then washed and incubated for 1 h at room temperature in an Alexa Fluor 546 conjugated secondary antibody (goat anti mouse Invitrogen). The cells were counterstained with DAPI (Sigma) and kept in the dark until images were taken using a microscope capable of detecting florescent images (Carl Zeiss Axio Imager I).

### Statistical analysis

All statistical analyses were performed using Graph Pad Prism software (Version 4, San Diego, CA). Comparisons between groups were made using independent *t*-test or analysis of variance (ANOVA), where appropriate. All data are presented as mean ± standard deviation of the mean (SD). Results were deemed significant when p < 0.05 (*), p < 0.01 (**) or p < 0.001(***).

## Results

### Hypoxia causes global DNA hypermethylation in human pulmonary fibroblasts

**Figure 1:**
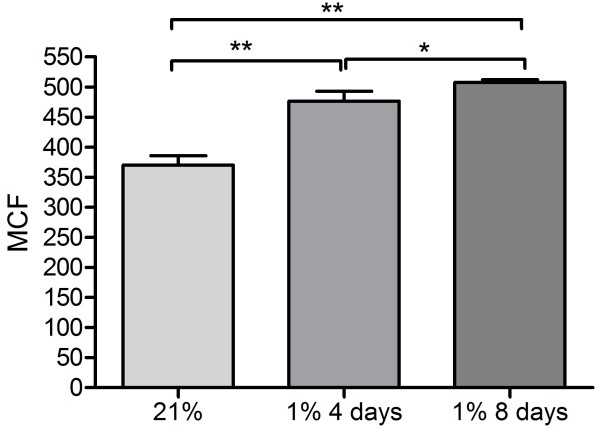
**Hypoxia causes global hypermethylation in human pulmonary fibroblasts.** CCD19Lu were grown for 4 and 8 days in 1% O_2_ and levels of DNA methylation were measured using an antibody directed towards 5 methylcytosine (5MeC) and quantitative flow cytometry. Hypoxic cells had significantly increased levels of 5MeC, measured as mean channel fluorescence (MCF), compared to levels in normoxic cells. Results are mean +/- SD for n = 3 experiments and statistical analysis was performed using one way anova where p < 0.05 (*) and p < 0.01 (**).

We and others have previously reported that hypoxia can alter the DNA methylation profile of tumour derived epithelial cells [7-9]. The impact of chronic hypoxia on global DNA methylation levels in normal human pulmonary fibroblasts was investigated using quantitative flow cytometry. Growing CCD19Lu in 1% O_2_ for 4 and 8 days significantly increased levels of 5MeC in these cells (Figure 1), a result that is in support of a role for hypoxia in modifying a cell’s epigenetic profile.

DNA methylation is involved in regulating gene expression, where an increased level of methylated cytosines in a gene promoter is associated with silencing of that gene. While a role for hypoxia in promoting myofibroblast differentiation has been acknowledged [25-28], more recently a role for DNA methylation in myofibroblast differentiation has also been proposed [13,14]. We therefore wished to investigate if the global hypermethylation in hypoxic pulmonary fibroblasts is associated with profibrotic phenotypic changes in the fibroblasts at similar hypoxic time points.

### Hypoxia increases Î±-SMA, collagen I and III in CCD19Lu

**Figure 2:**
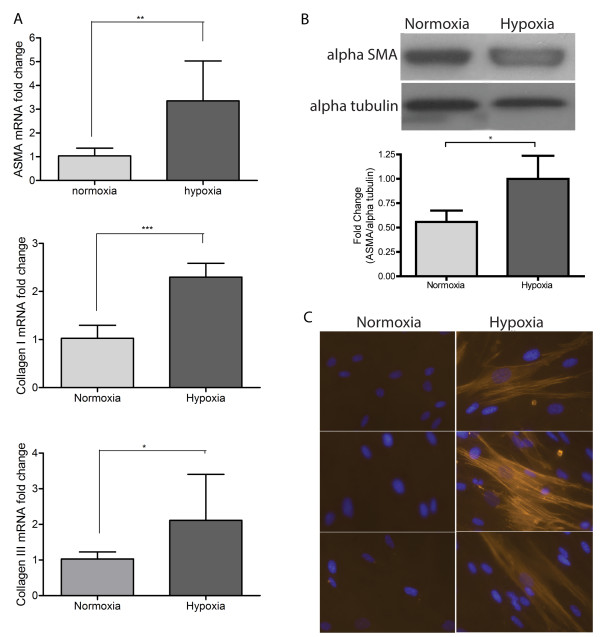
**Expression of myofibroblast markers are increased in hypoxic pulmonary fibroblasts.A**. Normoxic or hypoxic (1% oxygen, 8 days) pulmonary fibroblasts were analysed for mRNA expression of alpha smooth muscle actin (α-SMA), collagen I and collagen III using quantitative real-time PCR. Hypoxic cells expressed increased levels of α-SMA (p < 0.05), colIagen I (p < 0.001) and collagen III (p < 0.05) compared to normoxic controls. **B**. α-SMA protein expression was increased in hypoxic cells. This is a sample blot from n = 3 experiments. The increase in α-SMA was statistically significant when densitometry was performed. Results are mean +/- SD for n = 3 experiments. Students *t* test was used for statistical analysis. **C**. Immunocytochemistry for α-SMA (red) demonstrated that increased levels of the protein was present in hypoxic cells where it formed microfilaments. Cells were counterstained with nuclear specific DAPI (blue).

Hypoxia promotes myofibroblast differentiation. Pulmonary fibroblasts grown for 8 days in hypoxia expressed increased mRNA levels of α-SMA, collagen I and collagen III (Figure 2A). In addition α-SMA was increased at protein level (Figure 2B). Immunocytochemistry demonstrated that the increased α-SMA formed stress fibres consistent with myofibroblast morphology (Figure 2C).

Given that increased DNA methylation causes gene silencing, the increased expression of α-SMA, collagen I and III observed in hypoxic cells was not due to direct methylation of their promoters. Thus we investigated the expression of Thy-1, a gene whose expression is known to be regulated by DNA methylation [15]. Additionally Thy-1 (-ve) pulmonary fibroblasts are associated with a more differentiated myofibroblast phenotype [20-22]. Thus we hypothesised that Thy-1 expression may be regulated by DNA methylation in hypoxic CCD19Lu and this regulation contributed to a more fully differentiated myofibroblast like phenotype evident in the hypoxic cells.

### Hypoxia decreased expression of Thy-1

**Figure 3:**
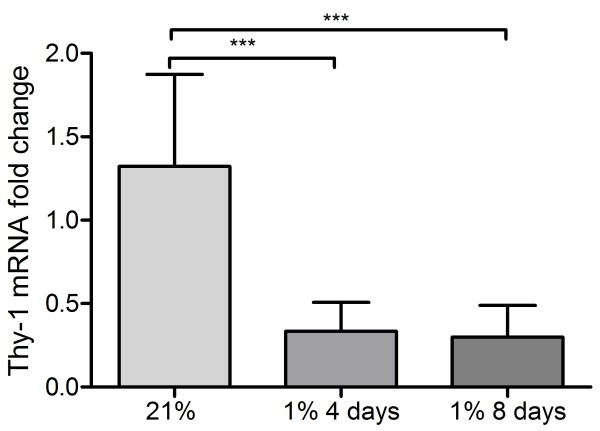
**Hypoxic human pulmonary fibroblasts express decreased levels of Thy-1 mRNA.** CCD19Lu were grown for 4 and 8 days in 1% oxygen and expression of Thy-1 was quantified using quantitative real time PCR. There was a significant reduction in Thy-1 expression in hypoxic cells after 4 and 8 days, p < 0.05 (*) and p < 0.01 (**), respectively, compared to normoxic control expression. Results are mean +/- SD for n = 4 experiments and students *t* test was used for statistical analysis.

**Figure 4:**
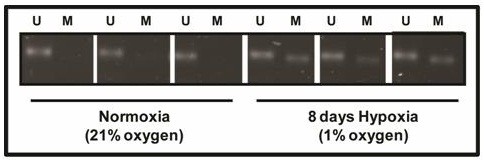
**The Thy-1 promoter becomes hemimethylated in hypoxic pulmonary fibroblasts.** Human pulmonary fibroblasts were grown in hypoxia for 4 and 8 days. Genomic DNA was bisulfite treated and methylation specific PCR (MSPCR) using unmethylated (U) and methylated (M) specific primers were used to investigate the methylation status of the Thy-1 promoter. MSPCR revealed Thy-1 promoter hemimethylation in hypoxic cells (n = 3) that was not present in normoxic controls (n = 3).

QPCR for Thy-1 revealed that hypoxia significantly reduced expression of the gene in fibroblasts after 4 and 8 days exposure to a 1% oxygen environment (Figure 3). In order to investigate if this reduction in gene expression was due to DNA hypermethylation in hypoxic CCD19Lu, two avenues of investigation were used to quantify Thy-1 promoter methylation. MSPCR revealed that the Thy-1 promoter became hemimethylated in 8 day hypoxic cell samples (Figure 4) suggesting that DNA methylation may be involved in reducing Thy-1 expression.

**Figure 5:**
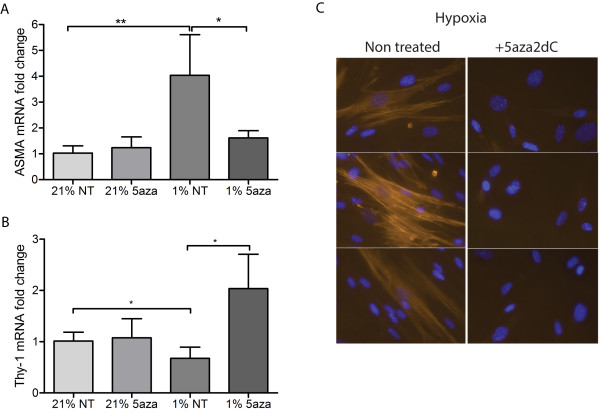
**The DNA hypomethylating agent 5-aza-2**^**′**^**-deoxycytidine restores mRNA expression of Thy-1 that is accompanied by a decrease in α-SMA expression.** Normoxic and hypoxic fibroblasts were treated with 1 μM 5-aza-2^′^-deoxycytidine (5-aza2dC) and quantitative real-time PCR was used to measure Thy-1 mRNA expression. **A**. 5-aza2dC inhibited hypoxia induced suppression of Thy-1 whereas in normoxia Thy-1 expression remained unchanged with 5-aza2dC treatment. **B**. Restoration of Thy-1 in 5-aza2dC treated hypoxic cells was accompanied by a reduction in α-SMA mRNA. **C**. Immunocytochemistry for α-SMA (red) demonstrated that restoration of Thy-1 using 5-aza2dC treatment decreased expression of α-SMA in hypoxic cells. Cells were counterstained with nuclear specific DAPI (blue). Results are mean +/- SD for n = 3 experiments and students *t* test was used for statistical analysis.

### The demethylating agent 5-aza2dC reversed hypoxic suppression of Thy-1 mRNA

Treating hypoxic fibroblasts with the DNMT inhibitor 5-aza2dC reversed hypoxic suppression of Thy-1 (Figure 5A) which provides further evidence that this gene is suppressed in hypoxia due to increased DNA methylation of its promoter. In addition, expression of Thy-1 in hypoxic 5-aza2dC treated samples was accompanied by reduced expression of mRNA α-SMA (Figure 5B) as well as reduced α-SMA protein expression (Figure 5C).

## Discussion

In support of other published research [7-9,29], the results presented here highlight hypoxia as an important regulator of a cell’s epigenetic profile. We show that chronic hypoxia induces a significant increase in global DNA methylation in human pulmonary fibroblasts that is associated with Thy-1 promoter methylation and the activation of a myofibroblast phenotype. These results are consistent with recent evidence of hypermethylation in tumour derived prostate epithelial cells exposed to chronic (>30 days) hypoxia [9]. In our study hypoxic human pulmonary fibroblasts had increased levels of 5MeC compared to normoxic controls although this change occurred at earlier hypoxic time points as the fibroblasts displayed a detectable increase in 5MeC as early as 4 days. Because hypermethylation was more pronounced at 8 days hypoxia we chose to conduct all further experiments at this time point. Given that at 4 days there were changes in global DNA methylation, it is likely that methylation events participate in myofibroblast differentiation at earlier hypoxic time points also, although this was not investigated further as part of this study.

Given the presence of tissue hypoxia in numerous disease processes, its modulation of epigenetic processes is an important observation. Global hypermethylation in hypoxic pulmonary fibroblasts is particularly significant given the fact that increased levels of global DNA methylation have been reported in IPF patient fibroblasts as well as in bleomycin treated mouse lung fibroblasts when their methylation status is compared to healthy control cells [13]. Indeed there is a growing body of evidence for a role for global epigenetic modifications in controlling the progression of myofibroblast differentiation [14]. In a folic acid-induced kidney fibrosis model, the DNMT inhibitor 5 azacytidine attenuated the disease [30], a further indication of the involvement of DNA methylation in fibrotic scar formation.

Gene silencing due to DNA methylation is thought to occur in one of two ways. Methylated DNA can directly impede transcription factor binding or alternatively methylated DNA attracts methyl binding domain (MBD) containing proteins, such as MeCP2 [31], which help promote a repressive chromatin environment by subsequent interaction between histone modifiers. This has been implicated in both pulmonary and liver fibrosis [32-34] and has been supported by MeCP2 knockout mice which display decreased levels of fibrosis compared to wild type models of both diseases [33,35]. Whilst indirect, the correlations between increased MeCP2 expression and worsening fibrosis also support a role for DNA hypermethylation in fibrotic disease pathogenesis.

Increased DNA methylation has the potential to induce phenotypic changes in a cell. Distinguishing them from fibroblasts, myofibroblasts express increased levels of mesenchymal markers such as α-SMA as well as synthesise significantly more extracellular matrix components like collagen I and III. Here we have demonstrated an association between DNA hypermethylation and an exaggerated myofibroblast phenotype in hypoxia. Previous groups have reported gene specific methylation events as contributing to such changes in pulmonary fibroblast phenotype. Huang et al. reported that the global hypermethylation in IPF patient myofibroblasts caused silencing of the prostaglandin receptor E2 (PTGER2) gene and proposed that methylation of this gene contributed to the myofibroblast phenotype [13] while Hagood et al. have provided evidence of Thy-1 methylation contributing to such differentiation [15]. In Thy-1 (-ve) rat lung fibroblasts treatment with a histone deacetylase inhibitor (HDACi) reactivated Thy-1 expression [23]. Interestingly other groups have also reported anti-fibrotic effects of HDACi on IPF fibroblasts [36]. Reports such as these suggest that histone modifications combined with alterations in DNA methylation participate to promote myofibroblast differentiation. It is therefore unlikely that methylation of one single gene results in a complete differentiation in cell phenotype, although it is plausible that silencing of one gene contributes to the progression of myofibroblast development.

Thus in globally hypermethylated hypoxic cells we chose to investigate the methylation status of Thy-1. The Thy-1 promoter contains a high frequency of CpG dinucleotides. Methylation of this region has previously been shown to control gene expression and Thy-1 has been reported as methylated in fibroblasts in fibrotic foci in IPF patient tissue [15]. Although *in vivo* hypoxia can reduce Thy-1 expression in mouse lung fibroblasts [24], methylation of the Thy-1 promoter has not been associated with hypoxia-induced suppression. Here we have demonstrated that methylation of Thy-1 occurs in hypoxic human fibroblasts and significantly reduces Thy-1 transcription which can be restored by treatment with the demethylating agent 5-aza2dC. Interestingly, 5-aza2dC had no effect on Thy-1 expression in normoxia while in hypoxic cells 5-aza2dC treatment resulted in a 2-fold increase in expression. Given that hypoxia can regulate chromatin remodelling [37], the addition of 5-aza2dC in this environment may further enhance transcription factor access to binding sites that were inaccessible in normoxia.

In this study we demonstrate that Thy-1 methylation is associated with differentiation towards a myofibroblast phenotype which is in accordance with previous evidence which has shown that there is a strong correlation between Thy-1 loss and increased expression of mesenchymal markers. Thy-1 (-ve) rat fibroblasts as well as Thy-1^-/-^ mice express increased mesenchymal markers like α-SMA even at basal levels when compared to expression in positive controls [22,24]. Thy-1^-/-^ mouse lungs also express increased basal levels of total collagen compared to wild type controls [24]. These observations are directly comparable to hypoxic fibroblast data shown here. Thy-1 suppression is accompanied by increased expression of α-SMA, collagen I and III. Loss of Thy-1 surface protein has previously been shown to increase activation of latent TGFβ1 [17,38] and this could be a mechanism involving Thy-1 by which hypoxia promotes myofibroblast differentiation.

The increase in mesenchymal markers in hypoxia suggests that the promoter regions of these genes do not become directly hypermethylated. It is therefore likely that hypermethylation of other genes contributes to the change in phenotype. The phenotype seen in hypoxia is indicative of a Thy-1(-ve) fibroblast and DNA methylation alterations, be it *via* regulation of Thy-1 or otherwise, are implicated in playing an important role in this change. In support of this, Thy-1 re-expression in hypoxic cells treated with 5-aza2dC exhibited a significant suppression of α-SMA gene and protein expression. Although we have not investigated any other gene specific methylation events it is likely there are other ‘anti-fibrotic’ genes that may be silenced by a similar mechanism. For example PGER2 and RASAL display anti-fibrotic effects and have previously been reported as methylated in myofibroblasts [13,30].

CCD19Lu are a commercially available human fibroblast cell line. These cells are not transformed and do senesce so should act as a normal pulmonary interstitial fibroblast. However, growing these cells on polystyrene plates has the potential to alter the cells phenotype. This is a limitation of this study and the use of primary human fibroblasts could reflect the cell type more accurately.

## Conclusion

These results implicate hypoxia as an important regulator of fibroblast phenotype by causing DNA hypermethylation in these cells. Hypoxic exposure was associated with the development of a myofibroblast. Additionally, we have shown that associated with these changes, the Thy-1 promoter becomes methylated in hypoxic fibroblasts resulting in reduced gene expression. We propose that this methylation event is likely to contribute to the observed change in phenotype. DNA methylation changes are likely important events in the pathogenesis of pulmonary fibrosis and understanding the mechanisms and effected genes in this process may in the future provide new therapeutic targets.

## Abbreviations

5MeC: 5 Methylcytosine; 5-aza2dC: 5-aza-2’-deoxycytidine; IPF: Idiopathic pulmonary fibrosis; DNMT: DNA methyltransferase; TGFβ: Transforming growth factor beta; α-SMA: Alpha smooth muscle actin; Thy-1: Thymocyte differentiation antigen-1; MSPCR: Methylation specific PCR.

## Competing interests

All authors declare no conflict of interest.

## Authors' contributions

CMR, CJW, RN and AL carried out the experiments and performed the statistical analysis reported in this study. CMR, CJW and JAB drafted the manuscript conceived of the study, participated in the design of the study and participated in its coordination. All authors read and approved the final manuscript.
